# 23327Enhanced photoelectric conversion efficiency of dye-sensitized solar cells by the incorporation of flower-like Bi_2_S_3_:Eu^3+^ sub-microspheres

**DOI:** 10.1038/srep23395

**Published:** 2016-03-21

**Authors:** Bingyu Xu, Guofeng Wang, Honggang Fu

**Affiliations:** 1 Key Laboratory of Functional Inorganic Material Chemistry, Ministry of Education, School of Chemistry and Materials Science, Heilongjiang University, Harbin, 150080, China

## Abstract

In this paper, TiO_2_-Bi_2_S_3_ and TiO_2_-Bi_2_S_3_:Eu^3+^ composite photoanodes were successfully designed, which can not only fully absorb visible light but also transfer the electron from Bi_2_S_3_ to TiO_2_ conduction band due to the narrow band gap and high conduction band of Bi_2_S_3_. Compared to pure TiO_2_ cell, the photoelectric conversion efficiencies of TiO_2_-Bi_2_S_3_ and TiO_2_-Bi_2_S_3_:Eu^3+^ composite cells were increased significantly. In addition, the efficiency of TiO_2_-Bi_2_S_3_:Eu^3+^ composite cells were higher than that of TiO_2_-Bi_2_S_3_ cell which could be attributed to the larger BET surface area of Bi_2_S_3_:Eu^3+^. The electron transport and interfacial recombination kinetics were investigated by the electrochemical impedance spectroscopy and intensity-modulated photocurrent/photovoltage spectroscopy. The results indicated that the interfacial resistance of the TiO_2_-dye|I_3_^−^/I^−^ electrolyte interface of TiO_2_-Bi_2_S_3_:Eu^3+^ composite cell was much bigger than that of pure TiO_2_ cell. In addition, the TiO_2_-Bi_2_S_3_:Eu^3+^ cell has longer electron recombination time and longer electron transport time than pure TiO_2_ cell. The charge collection efficiency of TiO_2_-Bi_2_S_3_:Eu^3+^ composite cell was higher than that of pure TiO_2_ cell.

In the past decade, dye-sensitized solar cells (DSSCs) have attracted extensive attention due to their easy fabrication, lowcost and relatively high conversion efficiency^1^,^2^. A typical DSSC consists of a dye-sensitized semiconductor photoanode, an electrolyte with the dissolved iodide/triiodide (I_3_^−^/I^−^) redox couple between the electrodes and a catalytic counter electrode^3^,^4^,^5^,^6^. Especially, an advanced photoelectrode is one of the most important prerequisites for highly efficient DSSCs. A variety of materials have been used as photoelectrodes and show good electrocatalytic activity. In general, TiO_2_ has been widely used as for DSSCs owing to their large surface area for loading more dye molecules, the sensitizer of the DSSC is mainly N719 dye, and the DSSC consists of TiO_2_ film sensitized by a dye for absorbing incident light. Recently, extensive studies on the individual components of DSSCs have been performed to further reduce production costs and to achieve better cell performance^7^,^8^,^9^. Many attempts have been made to enhance the performances of DSSCs by controlling the size, shape and morphologies of the semiconductors, utilizing low band gap organic materials, introducing buffer layers, and so on. Among them, one facile and efficient approach is to introduce foreign ions into organic semiconductors^10^,^11^. Some metal oxides^12^,^13^, nitrides^14^, carbides^15^ and sulfides^16^,^17^,^18^,^19^ have also been investigated as CEs due to their preferentially electrocatalytic activity. However, the application of sulfides as photoanode has been seldom reported. Metal sulfides gained more attention due to their facile preparation conditions and relatively low toxicity. As a well-known semiconductor, bismuth sulfide (Bi_2_S_3_) has the potential to improve the photocatalytic activity due to its narrow bandgap (~1.4 eV)^20^,^21^. It could has been used as a photoanode materials in DSSC due to its ability to absorb a large part of visible light up to 800 nm and transfer the electron from Bi_2_S_3_ to TiO_2_ conduction band. So far, there are many classic preparation methods for Bi_2_S_3_, such as hydrothermal method, solvothermal method, electrochemical deposition, microwave refluxing, organometallic complex decomposition and chemical vapor deposition^22^,^23^,^24^,^25^,^26^,^27^,^28^.

As a result, if one can design down-conversion luminescent TiO_2_-Bi_2_S_3_:Eu^3+^ composite photoanodes, not only the utilization of visible light can be improved but also the electron can transfer from Bi_2_S_3_ to TiO_2_ conduction band (see Fig. 1). And thus, the efficiency of the solar cells can be enhanced. In addition, metal ions doping semiconductor is also an effective strategy to improve the photocatalytic performance. Based on the consideration above, we report the synthesis of flower-like Bi_2_S_3_:Eu^3+^ through a hydrothermal route, and introduce Bi_2_S_3_:Eu^3+^ to the dye-sensitized solar photoanodes. The BET surface areas increased with increasing Eu^3+^ concentration. The photoelectric conversion efficiencies of TiO_2_-Bi_2_S_3_ and TiO_2_-Bi_2_S_3_:Eu^3+^ composite cells were significantly increased compared to pure TiO_2_ cell. The electron transport and interfacial recombination kinetics of cells were investigated in detail.

## Discussion

**Figure 2:**
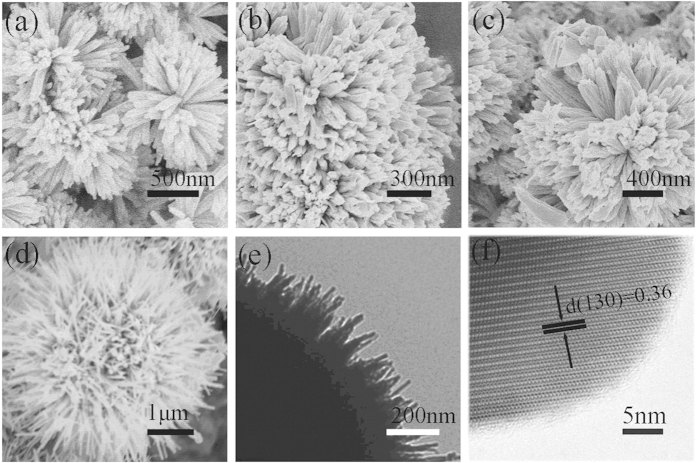
SEM (**a–d**), TEM and HRTEM (**e,f**) images of Bi_2_S_3_: Eu^3+^ nanocrystals prepared at 180 °C for 12h after annealing at 400 °C with different Eu^3+^ concentrations: (**a**) 5%, (**b**) 10%, (**c**) 15%, and (**d–f**) 20%.

Figure 2(a–d) represent the typical SEM images of the Bi_2_S_3_ products with different Eu^3+^ concentrations, which show that the products are composed of flower-like nanostructures. The average diameter of these superstructures is about 500 nm. The TEM and HRTEM images are presented in Fig. 2(e,f). The HRTEM image reveals that the interplanar spacing of 0.36 nm corresponds to the (130) plane of Bi_2_S_3_.

**Figure 3:**
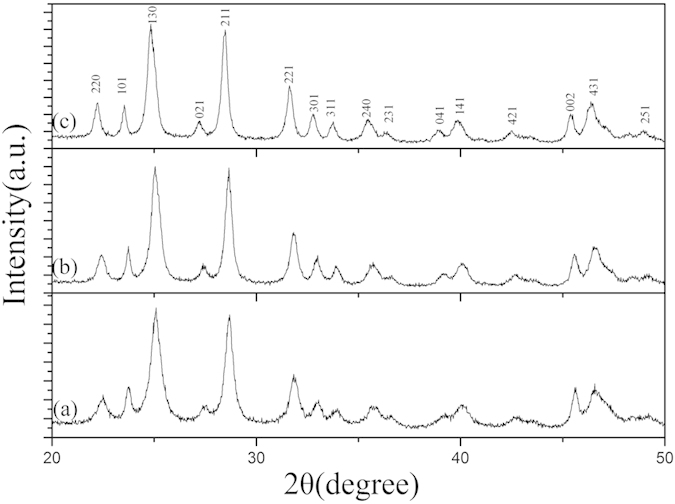
XRD patterns of Bi_2_S_3_: Eu^3+^ nanocrystals prepared at 180 °C for different reaction time: (**a**) 6 h, (**b**) 9 h, and (**c**) 12 h.

**Figure 4:**
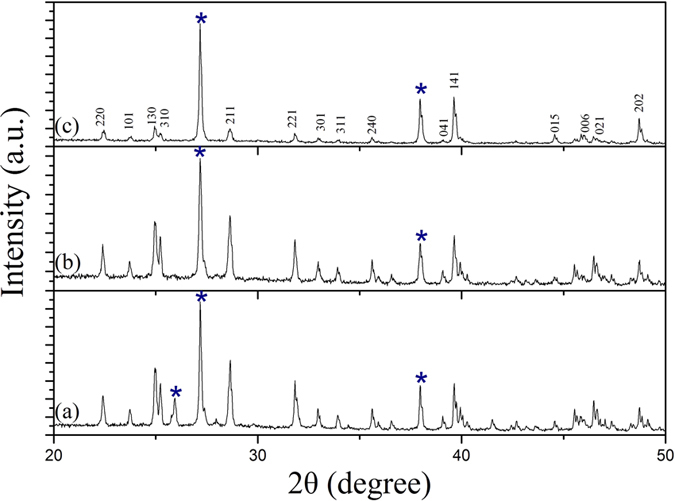
XRD patterns of Bi_2_S_3_: Eu^3+^ nanocrystals prepared at 180 °C for 12h with annealing at different temperatures for 2h: (**a**) 400 °C, (**b**) 600 °C, and (**c**) 800 °C.

**Figure 5:**
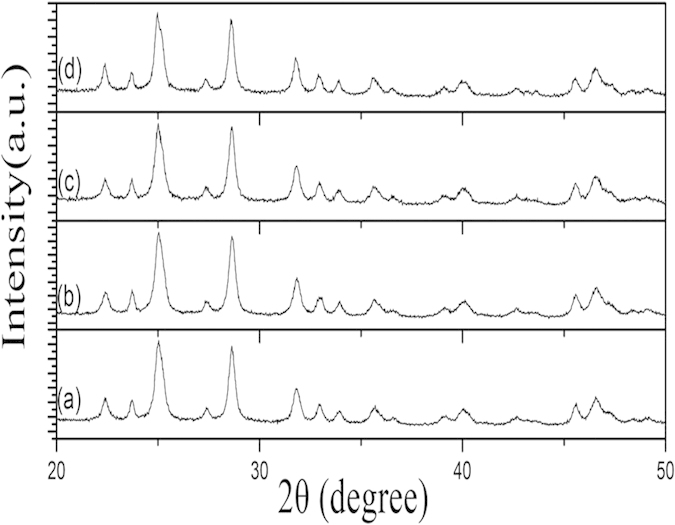
XRD patterns of Bi_2_S_3_:Eu^3+^ nanocrystals prepared at 180 °C for 12h with different Eu^3+^ concentrations: (**a**) 5%, (**b**) 10%, (**c**) 15%, and (**d**) 20%.

Figure 3 shows the XRD patterns of Bi_2_S_3_ nanocrystals (without annealing) with different reaction time, which are in good agreement with the standard data of orthorhombic phase Bi_2_S_3_ (JCPDS 17-0320). No other impurity peaks were detected. Figure 4 shows the XRD patterns of Bi_2_S_3_ nanocrystals after annealing at different temperatures. It can be seen crystalline size increases with increasing the annealing temperature. The peaks in Fig. 4 marked by asterisk (*) arise from cubic phase Bi particles (JCPDS 44-1256). The other diffraction peaks can be indexed to the orthorhombic phase Bi_2_S_3_. Figure 5 shows the XRD pattern of Bi_2_S_3_:Eu^3+^ nanocrystals (without annealing) with different Eu^3+^ concentrations. Obviously, no other impurity peaks were detected with increasing Eu^3+^ concentration.

**Figure 6:**
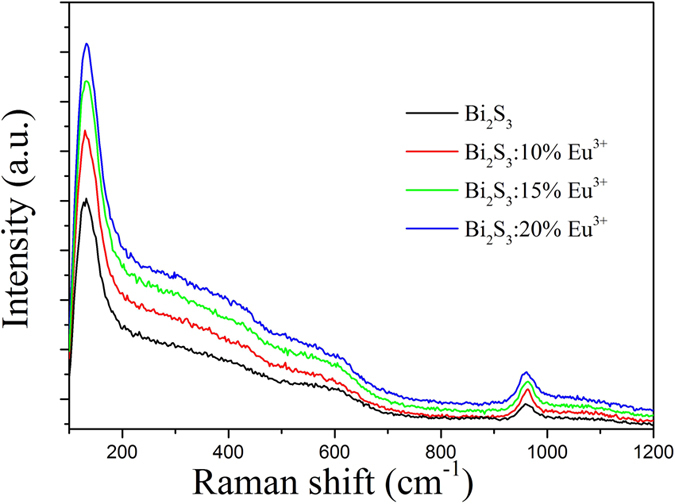
Raman spectra of Bi_2_S_3_:Eu^3+^ nanocrystals prepared at 180 °C for 12h with different Eu^3+^ concentrations.

Figure 6 shows the Raman spectra of Bi_2_S_3_:Eu^3+^ with different Eu^3+^ concentrations. The typical features in Raman spectra were located at 129 cm^−1^, 610 cm^−1^ and 965 cm^−1^.The 610 and 965 cm^−1^ bands are assigned to the Bi-S stretching vibrations. The 129 cm^−1^ is attributed to the surface of the optical phonon modes^29^.

**Figure 7:**
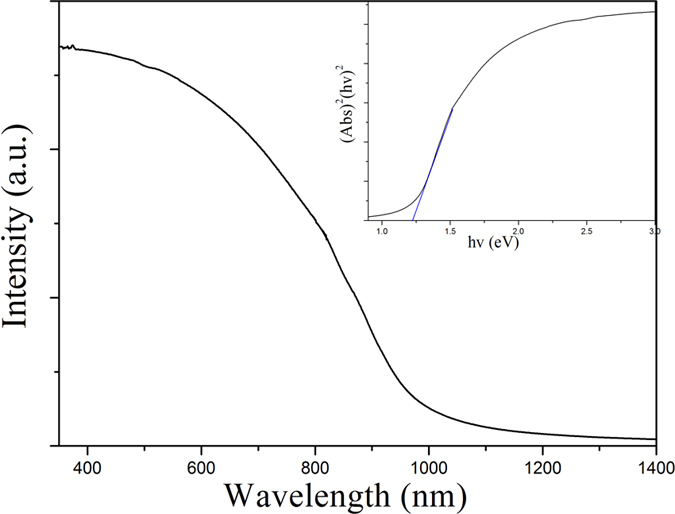
UV-Vis diffuse reflectance spectrum of Bi_2_S_3_ nanocrystals prepared at 180 °C for 12 h.

Figure 7 shows the UV-vis diffuse reflectance spectrum of Bi_2_S_3_ nanocrystals. The inset displays the plot of the transformed KubelkaeMunk function versus energy of light. The Kubelka-Munk function, F(R), allows the optical absorbance of a sample to be approximated from its reflectance: F(R) = (1 − R)^2^/2R. For a semiconductor sample this allows the construction of a Tauc Plot-(F(R).h)^n^ vs hv. For a direct band gap semiconductor the plot n = 1/2 will show a linear Tauc Region just above the optical absorption edge. Extrapolation of this line to the photon energy axis yields the semiconductor band gap. The calculated value of the band gap is about 1.22 eV for Bi_2_S_3_ nanocrystals.

**Figure 8:**
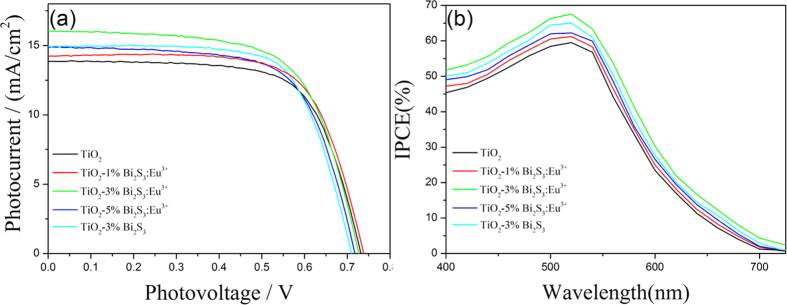
Photocurrent-voltage (I–V) curves (**a**) and IPCE (**b**) of TiO_2_, TiO_2_-Bi_2_S_3_, and TiO_2_-Bi_2_S_3_:Eu^3+^ cells with different concentration of Bi_2_S_3_:Eu^3+^.

In order to investigate the effects of TiO_2_-Bi_2_S_3_:Eu^3+^ on the photoelectric properties of DSSCs, the DSSC prototype devices were fabricated by using N719-sensitised TiO_2_-Bi_2_S_3_:Eu^3+^ composite electrodes. Figure 8(a) shows the photocurrent-voltage (I–V) curves of pure TiO_2_ cell, TiO_2_-Bi_2_S_3_ composite cell, and TiO_2_-Bi_2_S_3_:Eu^3+^ cells. The mass concentrations of Bi_2_S_3_:Eu^3+^ in the TiO_2_-Bi_2_S_3_:Eu^3+^ cells are 1%, 3%, and 5%, respectively. The corresponding values of the open-circuit voltage (V_oc_), short-circuit current (J_sc_), fillfactor (FF), and overall conversion efficiency (η), obtained from the curves of solar cells, are shown in Table 1. The result indicated that the photoelectric conversion efficiencies of the TiO_2_-Bi_2_S_3_ and TiO_2_-Bi_2_S_3_:Eu^3+^ composite cells were higher than that of pure TiO_2_ cell. The best photoelectric conversion performance was observed when the mass concentration of Bi_2_S_3_:Eu^3+^ was 3%. The high Voc of the TiO_2_-Bi_2_S_3_ could be attributed to heavy doping effects. Heavy impurity doping makes the conduction and valence bands shift, and brings about the so-called Band Gap Narrowing that resulting in the decrease of open circuit voltage. Figure 8(b) shows the incident photon to current (IPCE) spectra of pure TiO_2_, TiO_2_-Bi_2_S_3_, and TiO_2_-Bi_2_S_3_:Eu^3+^ composite cells. The results indicated that the photon-to-current conversion efficiency obviously increases by the incorporation of Bi_2_S_3_:Eu^3+^. With the increase of the proportion of Bi_2_S_3_:Eu^3+^ in TiO_2_-Bi_2_S_3_:Eu^3+^ cell, the efficiency increases first, and then decreases. At low concentrations of Bi_2_S_3_:Eu^3+^, the increase of the efficiency with the proportion of Bi_2_S_3_:Eu^3+^ could be attributed to the narrow bandgap and higher conduction band of Bi_2_S_3_, which not only improve the utilization of visible light but also transfer the electron from Bi_2_S_3_ to the conduction band of TiO_2_. However, the incorporation of Bi_2_S_3_:Eu^3+^ can influence the electrical conductivity of TiO_2_ and lead to a decrease in photocurrent. In addition, the effects of pure Bi_2_S_3_ on the photoelectric properties of DSSC were also studied. The results indicated that the photoelectric conversion efficiency of TiO_2_-Bi_2_S_3_ cell was lower than that of TiO_2_-Bi_2_S_3_:Eu^3+^ cell.

**Figure 9:**
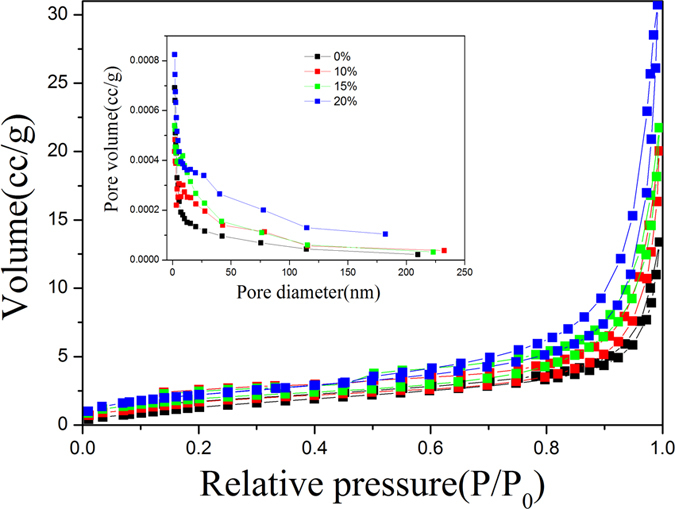
N_2_ adsorption-desorption isotherm curves and pore size distribution (inset) of Bi_2_S_3_:Eu^3+^ nanocrystals prepared at 180 °C for 12 h with different Eu^3+^ concentrations.

It is well known that the photoelectric performance was closely related to the ratios of the surface areas of samples. N_2_ adsorption-desorption isotherms and the corresponding BJH pore size distribution plots of the as-obtained Bi_2_S_3_:Eu^3+^ with different Eu^3+^ concentrations were performed to determine the surface area of the samples, as shown in Fig. 9. The BET surface areas are 4.8435, 5.4181, 6.6296, and 7.1739 m^2^/g for 0%, 10%, 15%, and 20% Eu^3+^, respectively.

**Figure 10:**
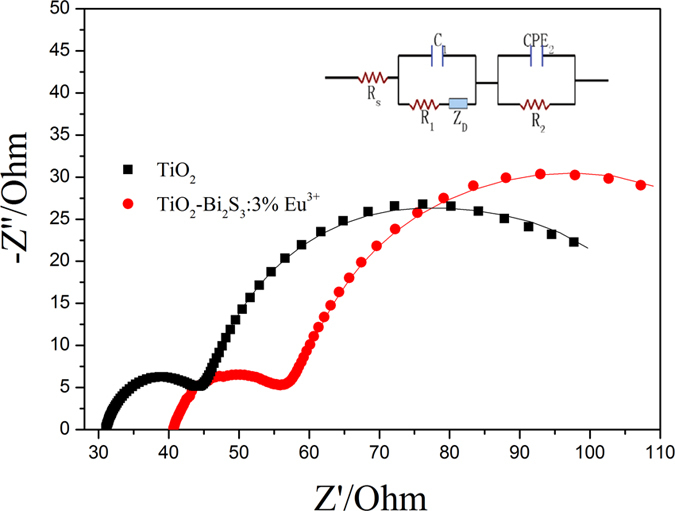
Nyquist plots of DSSCs comprised of pure TiO_2_ cell and TiO_2_-3%Bi_2_S_3_:Eu^3+^ cell. Inset is the equivalent circuit used to represent interfaces in composite solar cells composed of FTO|TiO_2_-dye|I^−^/I^3−^||Pt|FTO.

EIS is a powerful method to investigate internal resistances for the charge-transfer process of DSSCs. The wide frequency range of EIS means that it can measure wide-scale internal resistances of each electrochemical step at the same time^30^,^31^. DSSCs are complex systems which are composed of several interfaces. A high level of electron accumulation must occur because photogenerated electrons are not extracted immediately at the electrode contact under illumination. Generally, the impedance at low frequency (0.05–1 Hz) refers to the Nernst diffusion of I_3_^−^/I^−^ within the electrolyte. The impedance at high frequency (1–100 kHz) corresponds to the capacitance and charge-transfer resistance at the Pt^|^I_3_^−^/I^−^ electrolyte interface. The medium-frequency response at 1 Hz–100 Hz is related to the photoelectrode–dye|I_3_^−^/I^−^ electrolyte interface, where the accumulation of photoelectrons and redox shuttles is expected^32^,^33^. Figure 10 shows the EIS of pure TiO_2_ cell and TiO_2_-Bi_2_S_3_:Eu^3+^ cell. It can be seen that the interfacial resistance of the TiO_2_-dye|I_3_^−^/I^−^ electrolyte interface of TiO_2_-Bi_2_S_3_:Eu^3+^ cell is much bigger than that of pure TiO_2_ cell.

The inset in Fig. 10 shows the equivalent circuit fitting of the impedance spectra, R_s_[C_1_(R_1_O_1_)](R_2_CPE), which was used for all the DSSCs. R_s_ is the series resistance, corresponding to the sheet resistance of the FTO glass, the contact resistance and the wire resistance. R_2_ represents the charge transfer resistance between the photoelectrode-dye^|^I_3_^−^/I^−^ electrolyte interface. Z_Dif_ represents the finite-length Warburg impedance. The impedance of the finite-length Warburg diffusion is expressed as 
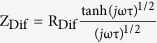
 where R_Dif_ = B/Y_0_, and τ = B^2^. B is a constant phase element.

According to the equivalent circuit, the EIS data obtained by fitting the impedance spectra of composite DSSCs are listed in Table 2. It can be seen that R_2_, representing the interfacial resistance of the TiO_2_-dye^|^I_3_^−^/I^−^ electrolyte interface, is 30.31 Ω for pure TiO_2_ cell and 41.30 Ω for TiO_2_-Bi_2_S_3_:Eu^3+^ composite cell. It is noted that the lower interfacial resistance can result in higher interfacial electron transfer, which is a beneficial factor for enhanced photoelectric conversion efficiency. In addition, the series resistance (R_S_) for pure TiO_2_ cell and TiO_2_-Bi_2_S_3_:Eu^3+^ cell are separately 31.53 Ω and 41.26 Ω, indicating that the incorporation of Bi_2_S_3_:Eu^3+^ is not beneficial for the interfacial electron transfer of FTO|TiO_2_.

**Figure 11:**
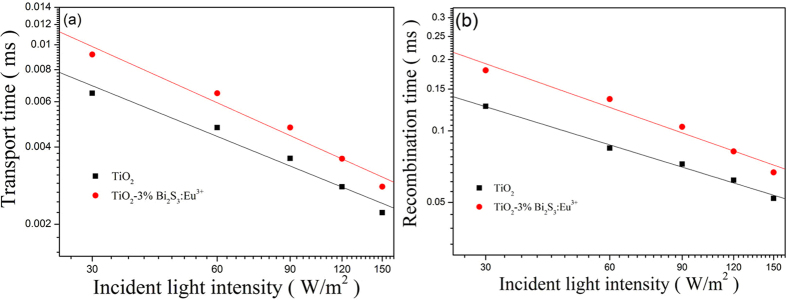
Comparison of (**a**) transport time constant and (**b**) recombination time constant for DSSCs comprised of pure TiO_2_ cell and TiO_2_-3%Bi_2_S_3_:Eu^3+^ cell.

In DSSCs, the electron recombination time (τ_n_), the electron transport time (τ_d_), and the charge collection efficiency (η_cc_) are important factors for the performance of DSSCs. Time-resolved photoluminescence spectrum can be used as an effective method to characterize the interface electron transport and electron recombination of the solar cell^34^,^35^. But restricted by the conditions, we can not test Time-resolved photoluminescence spectrum. However, the IMVS and IMPS are also a kind of effective characterization methods, which can be used to characterize the transmission life, charge separation and recombination of interface electrons. The IMPS response plots and IMVS response plots of pure TiO_2_ cell and TiO_2_-Bi_2_S_3_:Eu^3+^ composite cell are shown in Fig. 11. Compared with pure TiO_2_ cell, the TiO_2_-Bi_2_S_3_:Eu^3+^ composite cell has longer electron recombination time and longer electron transport time. It noted that longer transport time can result in poorer photoelectric properties, while longer recombination time is beneficial for enhancing photoelectric properties.

**Figure 12:**
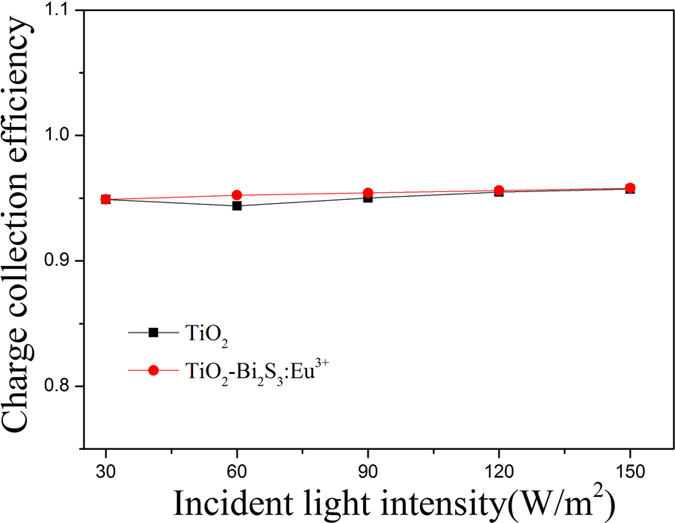
Comparison of charge collection efficiency for DSSCs comprised of pure TiO_2_ cell and TiO_2_-Bi_2_S_3_:Eu^3+^ cell.

The charge collection efficiencies (η_cc_) of DSSCs are determined by the relation: η_cc_ = 1 − τ_d_/τ_n_. Where, τ_d_ is a charge transport time and τ_n_ is a charge recombination lifetime. Figure 12 shows the charge collection efficiencies of pure TiO_2_ cell and TiO_2_-Bi_2_S_3_:Eu^3+^ cell. TiO_2_-Bi_2_S_3_:Eu^3+^ composite cell has a higher charge collection efficiency than pure TiO_2_ cell. All these results indicated that the performance of the solar cells can be improved by adding Bi_2_S_3_:Eu^3+^.

In summary, flower-like Bi_2_S_3_:Eu^3+^ was successfully prepared by a facial solvothermal method. The obtained Bi_2_S_3_:Eu^3+^ was chosen to design TiO_2_-Bi_2_S_3_:Eu^3+^ composite photoanodes. The result indicated that the photoelectric conversion efficiency were enhanced greatly by the incorporation of Bi_2_S_3_:Eu^3+^. The best photoelectric conversion performance was observed when the mass concentration of Bi_2_S_3_:Eu^3+^ was 3 wt%. The result of EIS analysis revealed that the interfacial resistance of the TiO_2_-dye^|^I_3_^−^/I^−^ electrolyte interface of TiO_2_-Bi_2_S_3_:Eu^3+^ composite cell was much bigger than that of pure TiO_2_ cell. In addition, the TiO_2_-Bi_2_S_3_:Eu^3+^ composite cell exhibited longer electron recombination time, longer electron transport time, and higher charge collection efficiency than those of pure TiO_2_ cell. Of course, the enhancement of the efficiency of the TiO_2_-Bi_2_S_3_:Eu^3+^ composite cells was also related to the larger BET surface areas of Bi_2_S_3_:Eu^3+^.

## Methods

### Synthesis of flower-like Bi_2_S_3_ nanocrystals

In a typical experiment, Bi(NO_3_)_3_, CH_4_N_2_S and Eu(NO_3_)_3_ were separated add to ethylene glycol (10 ml), and the solution was thoroughly stirred. Subsequently, the solution was transferred to a 50 ml Teflon-lined autoclave for 12 h at 180 °C. After cooling to room temperature, the final products were collected by means of centrifugation, washed with ethanol, dried at 80 °C in air and then annealed at different temperature.

### Fabrication of photoelectrodes

Fabrication of photoelectrode and the assembly of DSSCs: several pastes, from homogeneously mixing Bi_2_S_3_:Eu^3+^ and TiO_2_ (Degussa P25) into 3 mL of TiO_2_ colloid, were prepared with different mass concentrations of Bi_2_S_3_:Eu^3+^. The TiO_2_ colloid was prepared following the previously published synthesis procedure^36^. A screen-printed double layer of TiO_2_-Bi_2_S_3_:Eu^3+^ was used as the photoanode. The first layer of TiO_2_-Bi_2_S_3_:Eu^3+^ was prepared by a doctor-blade method on the FTO substrate and then sintered at 450 °C for 1 h. Subsequently, the second layer of TiO_2_-Bi_2_S_3_:Eu^3+^ was covered on the first TiO_2_-Bi_2_S_3_:Eu^3+^ film and then sintered at 450 °C for 30 min again. The sensitization of the photoelectrodes was achieved by immersing them into 0.5 mM ((C_4_H_9_)_4_N)_2_[Ru(4-carboxy-4′-carboxylate-2,2′ bipyridine)2(NCS)_2_] dye (N719, Solaronix SA, Switzerland) in acetonitrile and tertbutanol (volume ratio, 1:1) for 48 h at room temperature. The Pt counter electrodes were prepared following the previous literature^37^. The dye-sensitized photoanode was assembled with a Pt counter electrode into a sandwichtype cell. The sandwich-type cell was further fixed together with epoxy resin.The space between the electrodes was filled with the electrolyte, which comprised 0.6 M 1-propyl-2,3-dimethyl-imidazolium iodide, 0.05 M I_2_, 0.1 M LiI, and 0.5 M tert-butylpyridine (TBP) in 3-methoxypropionitrile (3-MPN), by capillary action.

### Materials Characterizations

The composition of the materials was determined by a Rigaku (Japan) D/MAX-rA X-ray diffraction meter (XRD) equipped with graphite monochromatized Cu Kα radiation (γ = 1.541874 Å), keeping the operating voltage and current at 40 kV and 40 mA. The size and morphology of the final products were investigated by scanning electron microscopy (SEM, Hitachi, S-4800) and transmission electron microscopy (TEM, JEOL, JEM-3010). UV-Vis absorption spectrum were determined by a UV–Vis spectrophotometer (Shimadzu UV-2550, Tokyo, Japan). The Raman spectra were measured by a HORIBA JOBIN YVON LabRam-HR 800 micro-Raman spectrometer.

### Photovoltaic properties study

Photovoltaic measurements were carried out with a solar simulator (Oriel, USA) equipped with an AM 1.5G radiation (1 sun conditions, 100 mW cm^−2^) filter was used as the light source. Current-voltage (J–V) curves were measured with a BAS100B electrochemical analyzer (Zahner Elektrik, Germany). The area of DSSCs is 1.5 cm^2^ and the irradiation area is 0.09 cm^2^ with a light intensity meter. The photoanode of Bi_2_S_3_:Eu^3+^ films were fabricated in the same condition. To make the data strictly and scientifically, all the cells was test for at least 5 times then obtained an average value. The EIS were performed with a computer-controlled IM6e impedance measurement unit (Zahner Elektrik, Germany) and carried out by applying sinusoidal perturbations of 10 mV with a bias of −0.8 V at a frequency ranges from 10 mHz to 1 MHz. The obtained spectra were fitted with ZsimpWin software in terms of appropriate equivalent circuits. The electron transport and recombination properties were measured by intensity-modulated photocurrent spectroscopy (IMPS) and intensity-modulated photovoltage spectroscopy (IMVS) (Zahner Elektrik, Germany). The DSSCs were probed through the photoanode side by a frequency response analyzer using a white lightemitting diode (wlr-01) as the light source. The frequency range was 0.1–1000 Hz. The irradiated intensity was varied from 30 to 150 mW cm^−2^.

## Additional Information

**How to cite this article**: Xu, B. *et al.* 23327Enhanced photoelectric conversion efficiency of dye-sensitized solar cells by the incorporation of flower-like Bi_2_S_3_:Eu^3+^ sub-microspheres. *Sci. Rep.* **6**, 23395; doi: 10.1038/srep23395 (2016).

## Figures and Tables

**Figure 1:**
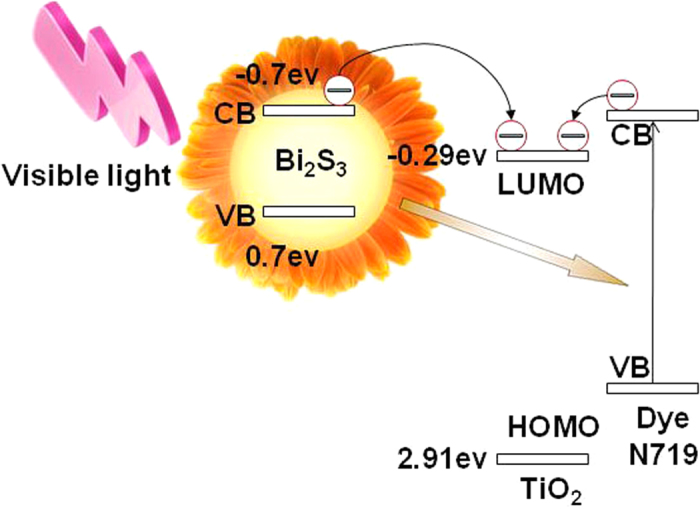
Mechanism of visible light harvesting in TiO_2_-Bi_2_S_3_:Eu^3+^ cells.

**Table 1:** Solar cell parameters of TiO_2_, TiO_2_-Bi_2_S_3_, and TiO_2_-Bi_2_S_3_:Eu^3+^ cells with different concents of Bi_2_S_3_:Eu^3+^.

Samples	I_sc_ (mA cm^−2^)	V_oc_ (V)	FF	η (%)
Pure TiO_2_	13.0056	0.743	0.68	6.95
TiO_2_-1%Bi_2_S_3_:Eu^3+^	14.2333	0.739	0.69	7.29
TiO_2_-3%Bi_2_S_3_:Eu^3+^	16.0667	0.727	0.65	7.47
TiO_2_-5%Bi_2_S_3_:Eu^3+^	14.8667	0.718	0.67	7.10
TiO_2_-3%Bi_2_S_3_	14.9333	0.711	0.69	7.33

**Table 2:** Parameters obtained by fitting the impedance spectra of composite solar cells using the equivalent circuit in the inset of Fig. 9.

DSSCs	R_s_/Ω	C_1_/F	R_1_/Ω	Y_o1_/S	B/s^1/2^	R_2_/Ω	CPE
Pure TiO_2_	31.53	9.378 × 10^−6^	12.00	0.01077	0.387	30.31	0.0005914
TiO_2_-Bi_2_S_3_:3%Eu^3+^	41.26	7.789 × 10^−6^	12.30	0.00641	0.252	41.30	0.00217
